# All-natural gelatin-based bioorthogonal catalysts for efficient eradication of bacterial biofilms†

**DOI:** 10.1039/d2sc03895a

**Published:** 2022-10-07

**Authors:** Ahmed Nabawy, Rui Huang, David C. Luther, Xianzhi Zhang, Cheng-Hsuan Li, Jessa Marie Makabenta, Vincent M. Rotello

**Affiliations:** Department of Chemistry, University of Massachusetts Amherst 710 N. Pleasant St. Amherst MA 01003 USA rotello@umass.edu

## Abstract

Bioorthogonal catalysis mediated by transition metal catalysts (TMCs) presents a versatile tool for *in situ* generation of diagnostic and therapeutic agents. The use of ‘naked’ TMCs in complex media faces numerous obstacles arising from catalyst deactivation and poor water solubility. The integration of TMCs into engineered inorganic scaffolds provides ‘nanozymes’ with enhanced water solubility and stability, offering potential applications in biomedicine. However, the clinical translation of nanozymes remains challenging due to their side effects including the genotoxicity of heavy metal catalysts and unwanted tissue accumulation of the non-biodegradable nanomaterials used as scaffolds. We report here the creation of an all-natural catalytic “polyzyme”, comprised of gelatin–eugenol nanoemulsion engineered to encapsulate catalytically active hemin, a non-toxic iron porphyrin. These polyzymes penetrate biofilms and eradicate mature bacterial biofilms through bioorthogonal activation of a pro-antibiotic, providing a highly biocompatible platform for antimicrobial therapeutics.

## Abstract 2

An all-natural biopolymer-based nanoemulsion catalyst for combatting MDR bacterial biofilms while maintaining excellent biocompatibility and biodegradability.

## Graphical abstract

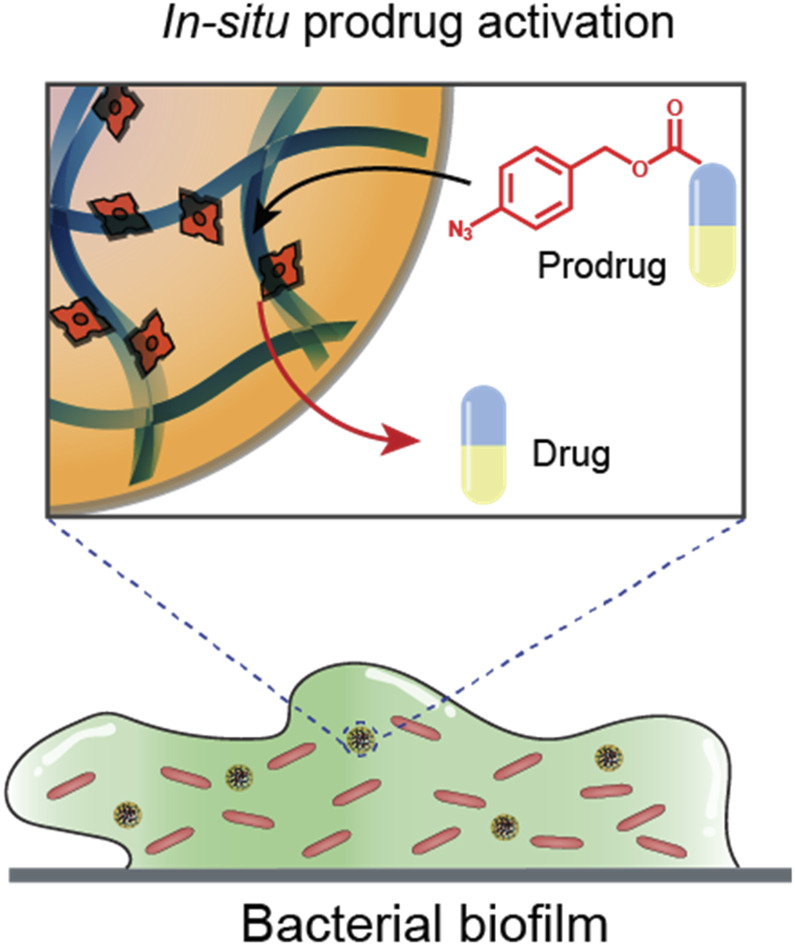

## Introduction

Bioorthogonal chemistry is a promising strategy for interrogating and modulating cellular bioprocesses, harnessing the toolkit of synthetic chemistry to perform transformations outside the capabilities of biological systems.^1–7^ Bioorthogonal uncaging reactions mediated by transition metal catalysts (TMCs) have the potential to revolutionize biomedicine through their ability to generate therapeutic agents locally, minimizing off-target effects.^8–11^ However, the direct use of ‘naked’ TMCs is challenging due to limitations of poor water solubility and catalyst deactivation in biological environments.^12^

Incorporating TMCs into nanoparticle hosts can solubilize and stabilize the catalysts, providing bioorthogonal ‘nanozymes’ that replicate structural and functional aspects of natural enzymes.^13,14^ These nanoscaffolds possess unique physicochemical properties that facilitate rational design and future applications.^15^ To date, a wide range of nanomaterials have been used to generate nanozymes, advancing the development of bioorthogonal catalysis.^16–21^ However, the common use of non-biodegradable inorganic nanomaterial scaffolds has concerns of unwanted tissue accumulation.^22,23^ Moreover, current nanozyme platforms rely heavily on the use of heavy metal catalysts such as Ru and Pd, resulting in the potential for long-term side effects including hepato-, geno-, and neurotoxicity.^24,25^

**Fig. 1:**
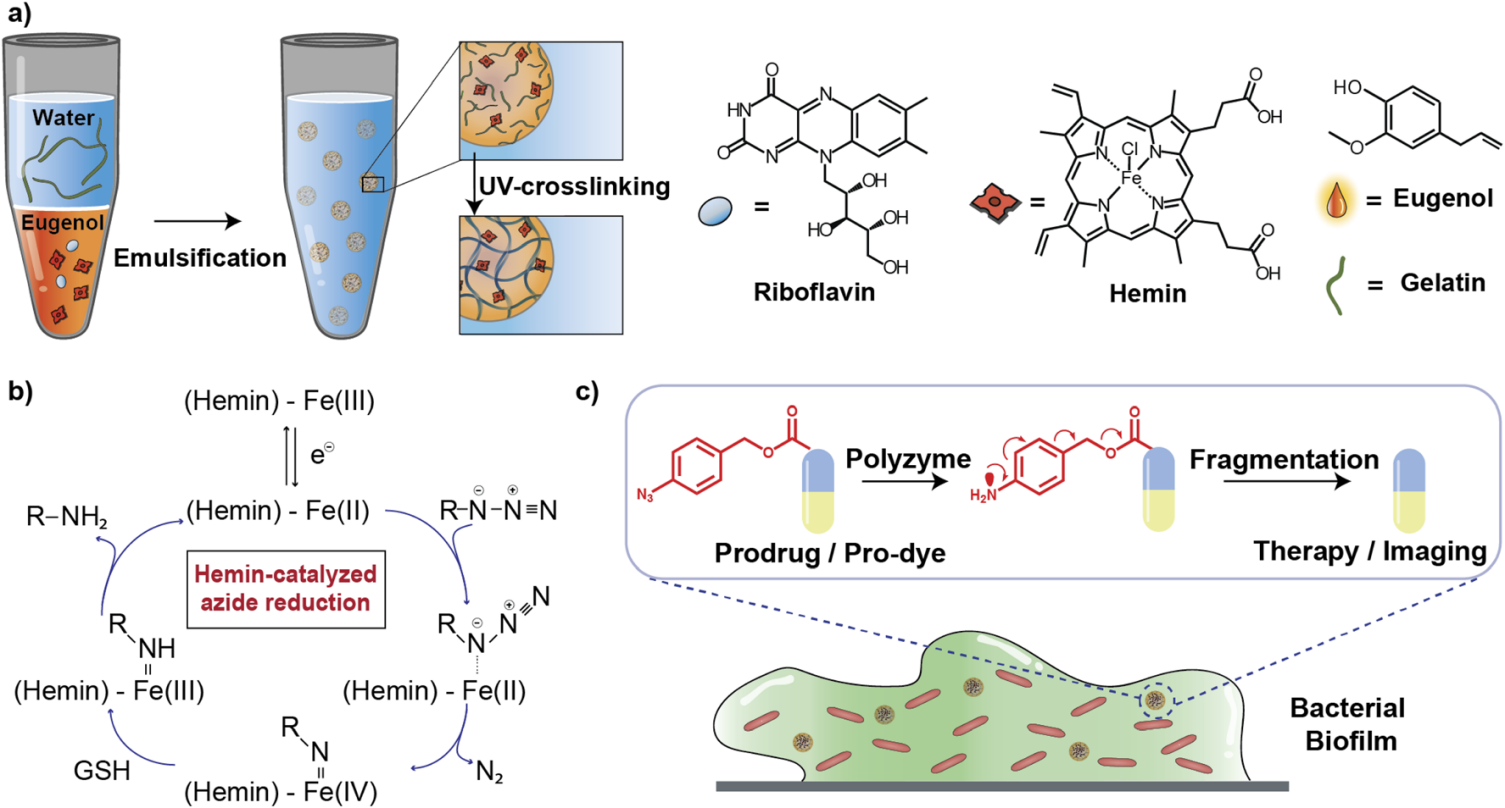
(a) Schematic demonstration of Fe_PZ fabrication. (b) Schematic demonstration of hemin-catalyzed azide reduction. (c) Schematic representation of the bioorthogonal activation within the biofilm matrix for imaging and therapeutics.

In this article, we describe the development of an all-natural biopolymeric ‘polyzyme’ nano-emulsion platform,^26^ aiming to provide a highly safe and efficient antimicrobial platform for potential clinical applications (Fig. 1). This nanoemulsion uses gelatin as a scaffold and eugenol (from clove oil)^27^ to provide a non-toxic hydrophobic phase for TMC encapsulation. This scaffold was then used to stabilize and solubilize the naturally occurring iron porphyrin hemin as the catalytic center. As a result of carefully choosing the materials, the polyzyme is inherently biocompatible, biodegradable, and non-cytotoxic. This Fe_PZ polyzyme was fabricated by dissolving hemin into eugenol, and emulsifying with dissolved riboflavin (vitamin B2) followed by UV exposure to provide stable photo-crosslinked nanoemulsions (Fig. 1a).^28^ The integration of the hemin catalyst into the highly modular and biodegradable nanoemulsion system affords an all-natural bioorthogonal polyzyme that is translatable and sustainable for biomedical applications. The potential therapeutic application of Fe_PZ was demonstrated through the efficient killing of bacteria in biofilms. Hemin-loaded nanoemulsions efficiently penetrated the biofilm and eradicated resident bacteria through *in situ* activation of a prodrug antibiotic, providing a promising strategy for the treatment of refractory biofilm infections.^29^ Taken together, this polyzyme platform utilizes all-natural components to install drug-activating ‘nanofactories’ inside biofilms, generating imaging and therapeutic agents and minimizing off-target effects.

## Results and discussion

### Generation and characterization of polyzymes

Fe_PZ polyzymes were generated by encapsulation of hemin into gelatin nanoemulsions (Fig. 1a).^26,30^ Eugenol serves as the interior oil phase of the nanoemulsions, providing a favorable environment for encapsulating hemin. Gelatin contains hydrophobic and hydrophilic amino acid domains that confer surfactant-like properties,^31^ allowing it to encapsulate the hydrophobic eugenol and the hemin as the TMC. Photoinitiated collagen cross-linking using riboflavin (vitamin B2) was adapted from a corneal collagen repair strategy,^32^ inducing gelatin fiber crosslinking and providing highly stable nanoemulsion catalysts (Fig. 1c).

Iron porphyrins catalyze the reduction of aryl azides to the corresponding amines in the presence of biogenic thiols, providing a robust and efficient strategy for uncaging reactions (Fig. 1b).^13^ Hemin, an endogenous iron-containing porphyrin was chosen as the catalytic center for the polyzymes.^33^ Hemin features high catalytic efficiency, biodegradability, and biocompatibility. Significantly, the hydrophobicity of the hemin catalyst facilitated partitioning into the eugenol phase of the nanoemulsion. Utilization of nature-derived components that are well-studied and known for their biocompatibility provides the potential for more rapid translation of these materials.^26^

**Fig. 2:**
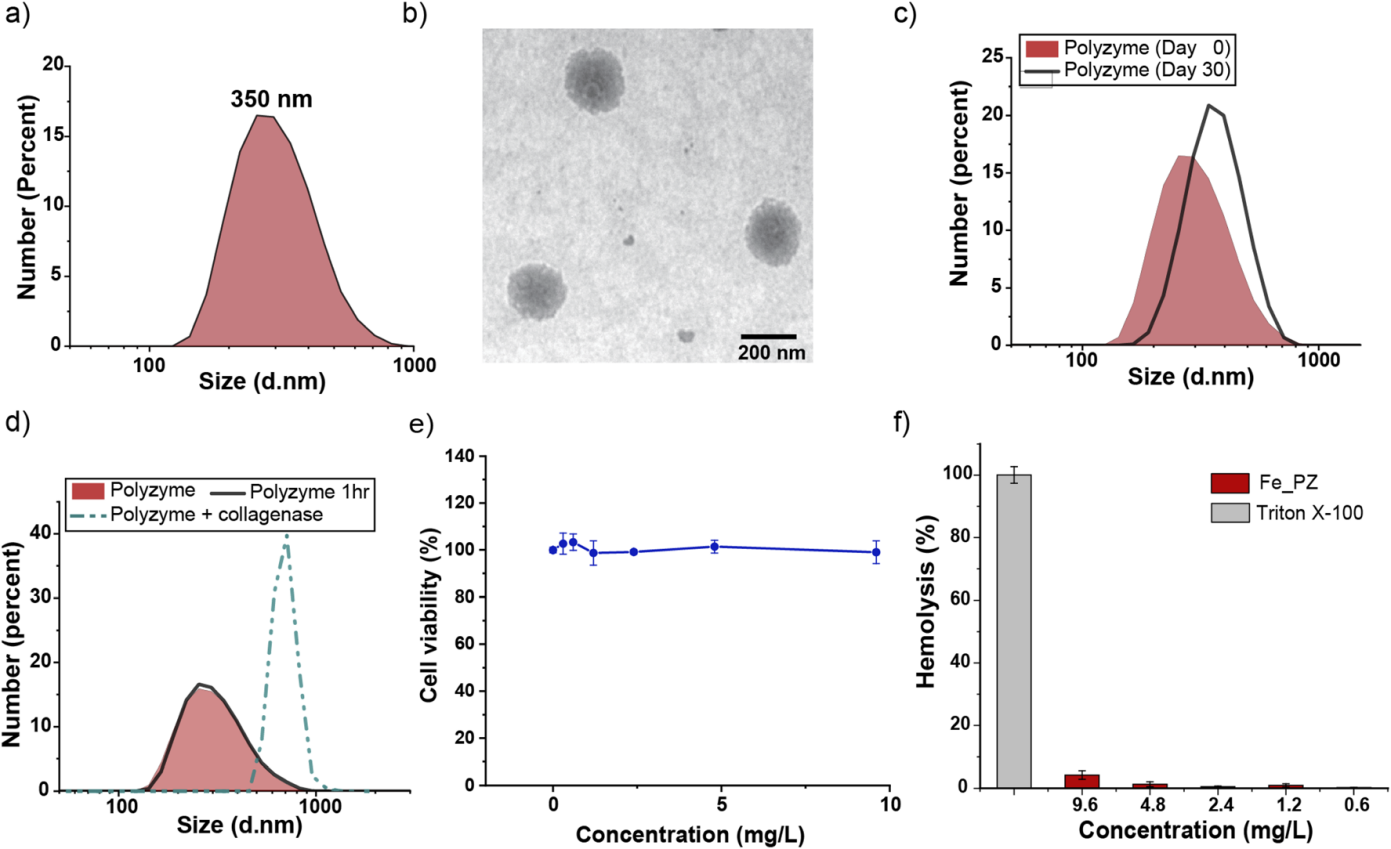
(a) Dynamic light scattering measurement (DLS) of Fe_PZ in phosphate-buffered saline (PBS, 150 mM). (b) Representative transmission electron microscopy (TEM) micrograph of Fe_PZ. (c) Fe_PZ show high stability for ≥30 days at room temperature. The polyzymes were stable in storage, with only a minimal change in DLS after 30 days. (d) Collagenase type I degrades Fe_PZ at 37 °C. The sharp increase in particle size after 1 h indicates the degradation of the polyzymes and subsequent aggregation of oil droplets. (e) Viability of 3T3 fibroblast cells after 24 h treatment with different concentrations of Fe_PZ (in the presence of GSH). (f) Hemolytic activity of Fe_PZ at different concentrations. Triton X-100 (0.1%) served as the positive control. Results demonstrate nonhemolytic nature of Fe_PZ at therapeutically relevant concentrations. Each experiment was performed in triplicate, and error bars represent standard deviation.

Fe_PZ polyzymes were fabricated by emulsifying a suspension of eugenol (3 μL oil loaded with 1 mg mL^−1^ riboflavin and 17.2 mg mL^−1^ hemin) into an aqueous solution of gelatin (Fig. 1a). Next, UV irradiation (365 nm) was employed to initiate covalent crosslinking of the gelatin matrix, generating stable nanoemulsions. Dynamic light scattering (DLS) measurements showed that the size of Fe_PZ is ∼350 nm with a narrow size distribution (polydispersity index = 0.023) (Fig. 2a). This particle diameter is generated under a range of conditions, and was hence used for further studies. The amount of hemin encapsulated into the nanoemulsions was quantified using UV-vis spectroscopy, with 0.43 mg catalyst per mg gelatin (Fig. S1†).

Transmission electron microscopy (TEM) micrographs indicated a spherical morphology for Fe_PZ (Fig. 2b). The size of Fe_PZ revealed by TEM (∼200 nm) is smaller than observed by DLS, presumably due to partial collapse of the polyzymes upon removal of oil core. After characterization of the size and morphology of the polyzymes, we examined the colloidal stability and degradability of the polyzyme platform *via* monitoring changes in particle size by DLS. Fe_PZ remained stable at room temperature for at least 30 days in solution (Fig. 2c). However, these nanoemulsions degraded within an hour in the presence of collagenase I (Fig. 2d).

Next, we investigated Fe_PZ biocompatibility using mammalian NIH 3T3 fibroblasts, chosen for the important role fibroblast cells play in the wound healing process.^34^ Fibroblasts were incubated with different concentrations of Fe_PZ (in the presence of glutathione (GSH) for 24 h). Then, cell viability was quantified using Alamar Blue assay. As shown in Fig. 2e, Fe_PZ showed no acute cytotoxicity at therapeutically relevant concentrations. Moreover, Fe_PZ demonstrated no hemolytic activity against human red blood cells at highest tested concentration of 9.6 mg L^−1^, indicating excellent biocompatibility of this nanoemulsion catalyst (Fig. 2f).

### Catalytic efficiency of polyzymes in solution

**Fig. 3:**
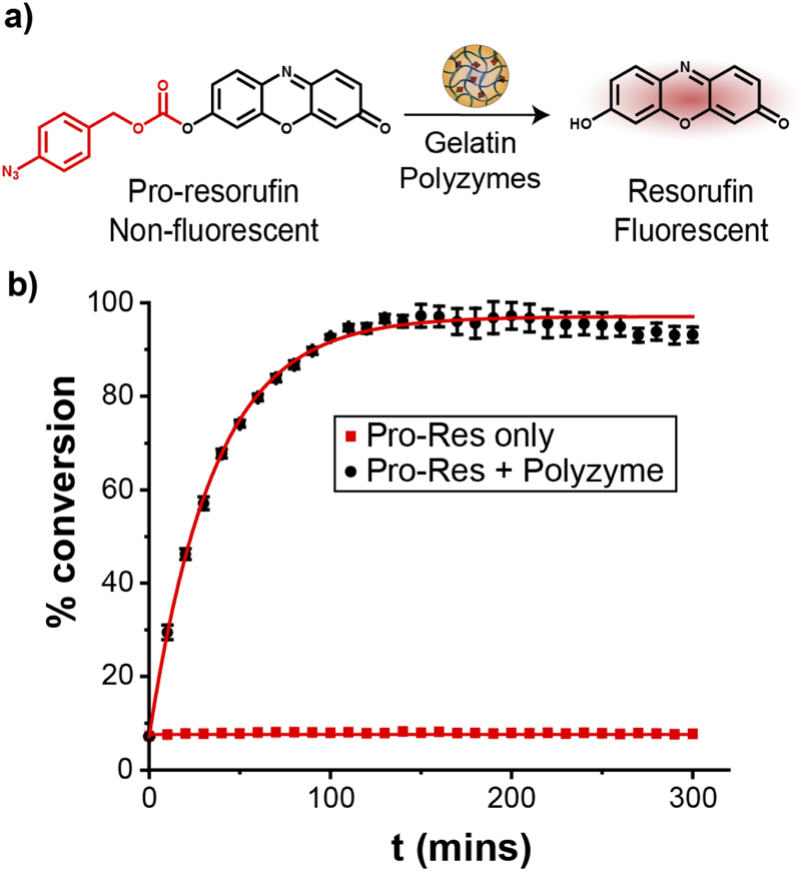
(a) Bioorthogonal uncaging of aryl azide-protected resorufin. (b) Catalytic activity of Fe_PZ was tracked by measuring changes in fluorescence (ex. 560 nm, em. 590 nm) of pro-resorufin solutions over time. Glutathione (GSH, 1 mM) was used as a cofactor for redox cycling. The curves are fit with a pseudo-first order kinetic model.

The catalytic efficiency of Fe_PZ was quantified by uncaging a nonfluorescent resorufin-based pro-fluorophore (pro-Res, Fig. 3a). Masking the phenolate group of resorufin with an aryl azide carbonate unit rendered the dye non-fluorescent. After the hemin-induced catalytic reduction of the aryl azide, pro-Res was transformed into strongly fluorescent resorufin. A rapid increase in resorufin fluorescence was observed after the addition of the polyzymes (4.8 mg L^−1^) to the pro-Res in M9 media at 37 °C, while no significant change in fluorescence occurred in pro-Res only (Fig. 3b). In contrast, free hemin catalysts at the same concentration suffered a >70% loss of activity (Fig. S2†). For these studies, glutathione (1 mM) was used as a reductant for reductive recycling of the catalyst. These results indicated that the essential oil core of polyzymes solubilize and shield the hydrophobic TMCs allowing higher catalytic efficiency at lower, and potentially safer, concentrations. The turnover number (TON) of the polyzyme was 0.014 s^−1^ on a per polyzyme basis (Fig. S3†).

### Penetration of gelatin polyzymes into biofilms

After probing the catalytic properties of gelatin polyzymes in solution, we next studied bioorthogonal catalysis in biofilms. Biofilm-associated infections are responsible for several chronic diseases, including wound infections, endocarditis, osteomyelitis, and implant dysfunction.^29,35^ The extracellular polymeric substance (EPS) matrix of biofilms serves as a physical barrier to most hydrophobic molecules, rendering biofilms challenging to treat.^36^

**Fig. 4:**
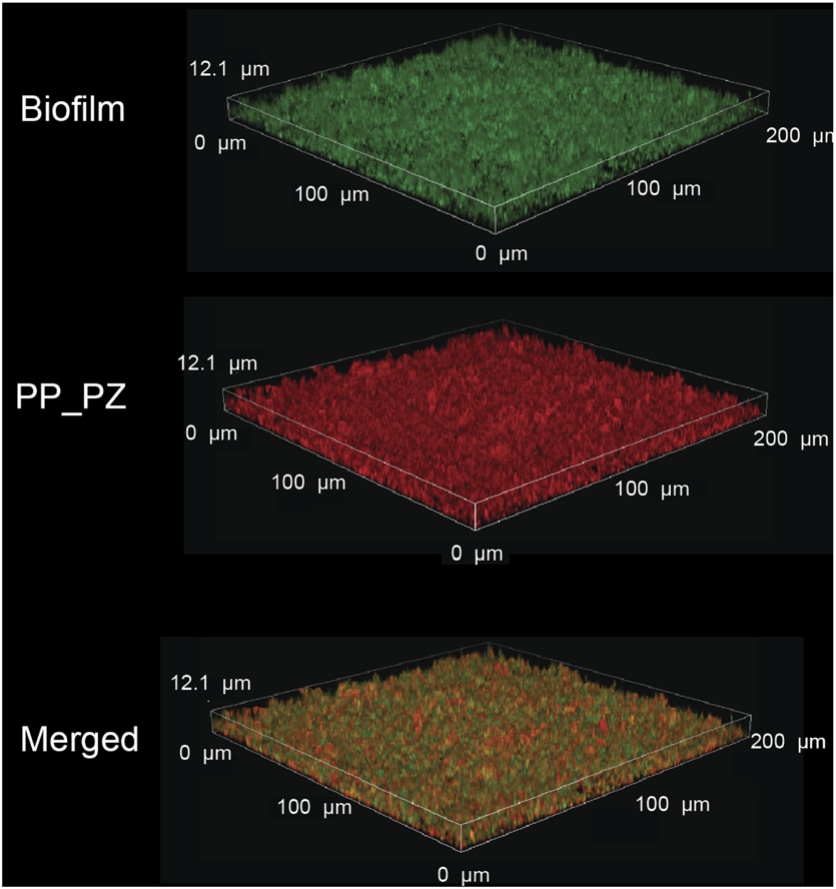
PP_PZ penetration into GFP-expressing *E. coli* biofilms after incubation for 2 h in M9 media, as monitored by confocal microscopy. The iron-free hemin equivalent Protoporphyrin IX was used to provide a fluorescent analog for imaging.

The ability to transport hydrophobic hemin catalyst into biofilms is essential for bioorthogonal activation of imaging agents and therapeutics.^13^ We first used confocal laser scanning microscopy to monitor the penetration and distribution of polyzymes into biofilms. Gelatin is cationic at low pH levels found in biofilms^37,38^ and it is expected that this positive charge would enable transport of the polyzyme into biofilms.^39–42^ Since hemin is non-fluorescent, we replaced the encapsulated hemin with the de-metallated analog protoporphyrin IX to provide PP_PZ that exhibits red fluorescence. PP_PZ was incubated with mature 4 day old biofilms of green fluorescent protein (GFP)-expressing *E. coli*. As shown in Fig. 4, gelatin polyzymes penetrated completely into the biofilm matrix, with full co-distribution of porphyrin and bacteria GFP signals. These results indicate that the gelatin-based nanoemulsion transported hydrophobic catalysts deep into biofilms for imaging and therapeutic purposes.

### Catalytic efficiency of gelatin polyzymes inside biofilms

Having demonstrated penetration of the polyzymes into biofilms, we next determined their bioorthogonal catalytic properties in biofilms through activation of pro-Res, as visualized by confocal microscopy. First, Fe_PZ (4.8 mg L^−1^) polyzymes were incubated with mature 4 day old biofilms of green fluorescent protein (GFP)-expressing BL21 *E. coli* for 2 h to allow biofilm penetration. Bacterial biofilms were then washed with PBS three times to remove any excess polyzyme.

**Fig. 5:**
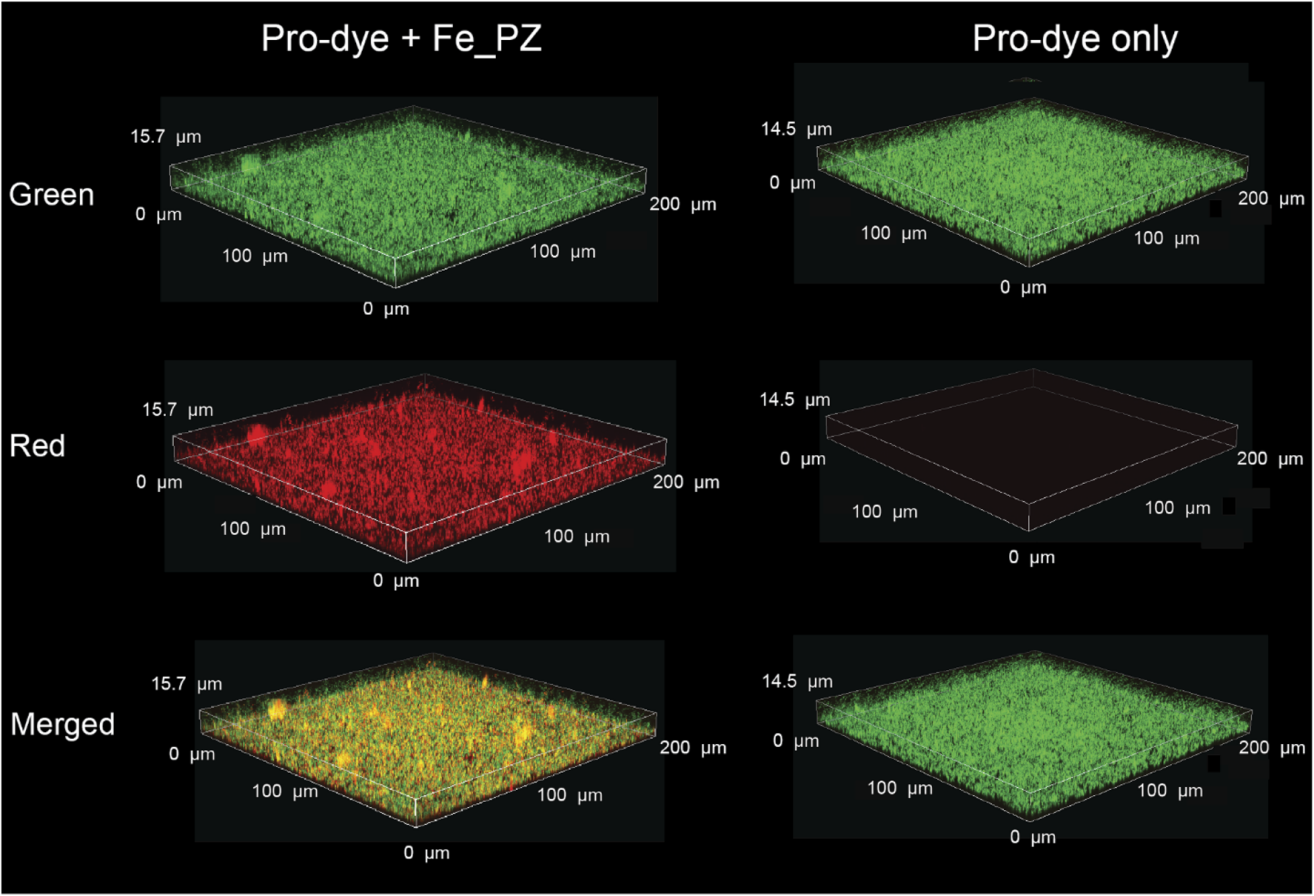
Catalytic activity of Fe_PZ in bacterial biofilm was monitored by confocal microscopy. Green fluorescent protein-expressing BL21 *E. coli* biofilms were treated with polyzyme (2 h, 4.8 mg L^−1^) followed by incubation with resorufin-based pro-dye (1 h, 5 μM); negative control is biofilms incubated with pro-dye only. GSH (1 mM) was used as a cofactor.

*In situ* bioorthogonal catalysis was then established by incubating the biofilms with pro-Res (5 μM) and GSH (1 mM) in M9 media for 1 h. As shown in Fig. 5, the GFP-expressing *E. coli* can be observed in the green channel. The bright red fluorescence indicates that in the presence of polyzyme pro-Res was activated in biofilms. Biofilms incubated with pro-Res only exhibited negligible red fluorescence, demonstrating the biorthogonality of pro-Res uncaging. The merged channels demonstrated the colocalization of the generated red resorufin with GFP-expressing *E. coli*, indicating that the uncaged resorufin was fully distributed throughout the mature biofilm matrix.

### Antimicrobial activity through activation of pro-antibiotics using polyzymes

**Fig. 6:**
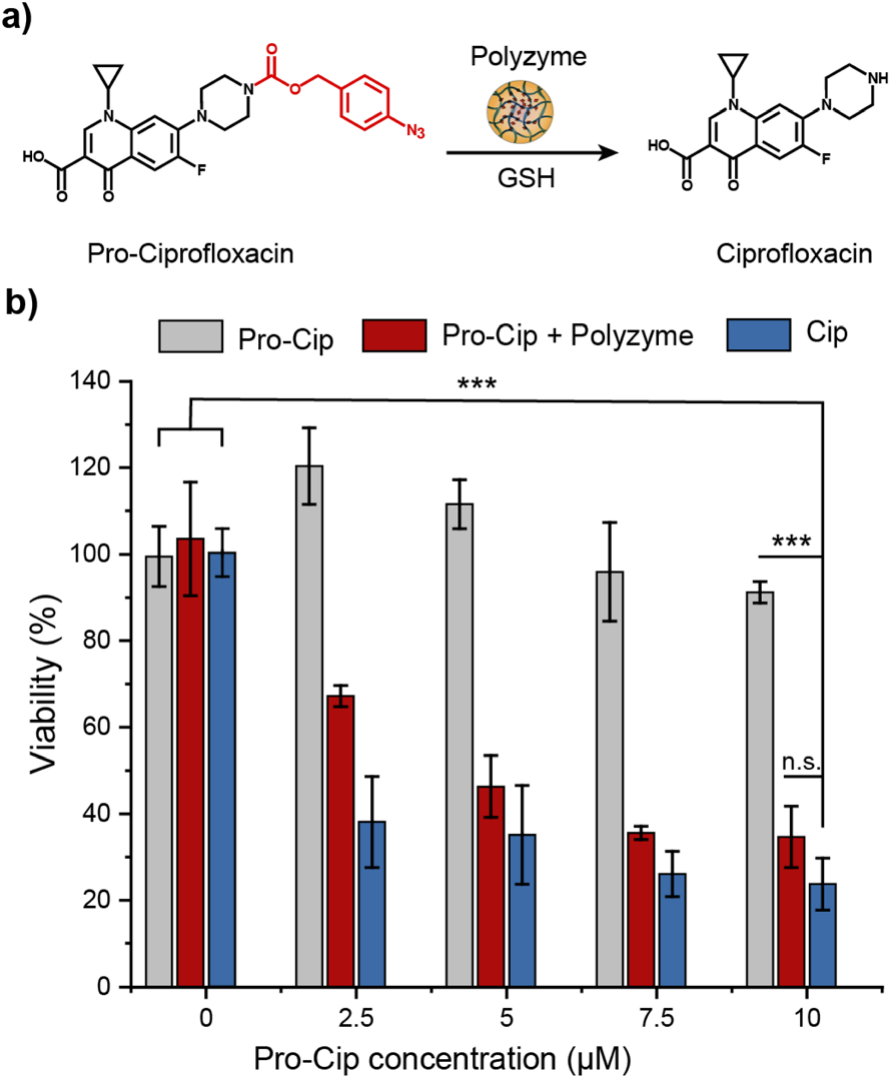
(a) Schematic representation of the activation of pro-Ciprofloxacin. We used 1 mM GSH as the cofactor. (b) Viability of *E. coli* (CD-2) biofilms incubated with Fe_PZ (2 h, 4.8 mg L^−1^) followed by treatment with prodrugs (6 h). Biofilms treated with prodrug, drug, and Fe_PZ (4.8 mg L^−1^) were used as controls. Each experiment was performed in triplicate, and error bars represent standard deviation. Statistical analysis was done using one-way analysis of variance (ANOVA). ***p < 0.001.

The therapeutic potential of Fe_PZ was demonstrated *via* the activation of an antibiotic prodrug. The widely used fluoroquinolone antibiotic ciprofloxacin (Cip) was chosen as the antimicrobial agent due to its broad-spectrum activity.^43^ The pharmacophore of this antibiotic was blocked through functionalization with a bulky aryl-azide carbamate moiety on its secondary amino group, generating a prodrug (pro-Cip).^44^ This caging strategy prevents Cip from binding to its target bacterial enzymes, DNA gyrase and topoisomerase IV,^45^ diminishing antimicrobial activity by more than two orders of magnitude (Fig. S4†). Pro-Cip was uncaged through bioorthogonal catalysis by polyzymes, releasing the active antimicrobial agent (Fig. 6a).

The efficacy of pro-Cip activation was established using mature 4 day old pathogenic *E. coli* (CD-2) biofilms. These biofilms were first incubated with Fe_PZ for 2 h to allow sufficient biofilm penetration. Next, the media was removed, and biofilms were washed three times with PBS followed by incubation with pro-Cip and GSH. Control conditions were provided by pro-Cip alone (negative) and Cip alone (positive) respectively. After incubation for 6 h, biofilm viability was quantified using an Alamar Blue assay. As shown in Fig. 6b, biofilms incubated with Fe_PZ and pro-Cip demonstrated dose-dependent biofilm killing, with efficacy approaching that of ciprofloxacin. As expected, biofilms incubated with either polyzyme or pro-Cip alone showed no decreased viability at the tested concentrations, demonstrating the biorthogonality of the activation. Significantly, this therapeutic strategy combining Fe_PZ and pro-Cip showed no acute cytotoxicity at therapeutically relevant concentrations (Fig. S5†). Given that Fe_PZ can eliminate pathogenic biofilms without harming mammalian cells, these results demonstrate the potential of the polyzyme platform for treatment of wound biofilm infections.

## Conclusions

In summary, bioorthogonal polyzymes were generated using all-natural components: hemin as catalyst, encapsulated in a gelatin–eugenol nanoemulsion stabilized through riboflavin-mediated crosslinking. These nanoemulsions were stable in solution and readily biodegraded enzymatically by collagenase. The integration of the hemin catalyst into the nanoemulsion system enhanced the stability and water solubility of the catalyst while preserving its catalytic activity in solution and in the complex environment of biofilms, as demonstrated through pro-fluorophore activation. These polyzymes (Fe_PZ) were likewise promising therapeutic systems, effectively penetrating into biofilms and uncaging pro-antibiotics *in situ*. Future studies will focus on utilizing the polyzyme platform for *in vivo* antimicrobial therapy. Taken together, this bioorthogonal all-natural polyzyme platform provides on-demand drug ‘nanofactories’ for treating biofilm-associated infections. More broadly, these studies present a strategy for maximizing therapeutic efficacy and minimizing side effects for a broad range of disease targets.

## Data availability

All supporting data is provided in the ESI.†

## Author contributions

A. Nabawy and R. Huang led the conceptualization, investigation and data analysis of the work and the writing of the original draft. D. C. Luther, X. Zhang, C. H. Li and J. M. Makabenta supported the investigation and data analysis of the work. V. M. Rotello helped the supervision of the work and led the review and editing of the draft.

## Conflicts of interest

There are no conflicts to declare.

## Supplementary Material

SC-013-D2SC03895A-s001
