# Supplementary material for: All-natural gelatin-based bioorthogonal catalysts for efficient eradication of bacterial biofilms
Source: Chem Sci. 2022 Oct 7;13(41):12071–7. doi: 10.1039/d2sc03895a (PMC9600305; doi:10.1039/d2sc03895a)
Supplement: SC-013-D2SC03895A-s001 [file SC-013-D2SC03895A-s001.pdf]

Electronic Supplementary Material (ESI) for Chemical Science.
This journal is © The Royal Society of Chemistry 2022

Supplementary Information

All-natural Gelatin-Based Bioorthogonal Catalysts for Efficient Eradication of Bacterial Biofilms

Ahmed Nabawy[§], Rui Huang[§], David C. Luther, Xianzhi Zhang, Cheng-Hsuan Li, Jessa Marie Makabenta and Vincent M. Rotello[*]

Department of Chemistry, University of Massachusetts Amherst, 710 North Pleasant Street, Amherst, Massachusetts 01003, United States

§ These authors contributed equally

*E-mail: rotello@chem.umass.edu

Table of Contents

1. Materials 2
2. Preparation of gelatin polyzymes: 2
3. Size of polyzymes 2
4. Quantification of hemin loaded in polyzymes 2
5. Catalytic activity of Free Hemin catalysts 3
6. Kinetic behavior of gelatin-based polyzymes 3
7. Minimum biofilm inhibitory concentration (MBIC) of Cip and pro-Cip 4
8. Biocompatibility of polyzyme and prodrug in mammalian cells 5
9. Activation of pro-dye and prodrug in bacterial biofilms 5
10. Synthesis of pro-resorufin and pro-ciprofloxacin 5

1. Materials

All chemicals and solvents for syntheses were purchased from Fisher Scientific and Sigma-Aldrich, and used without further purification, unless otherwise stated. The chemicals were used as received. All reagents and solvents were purchased from Fisher Scientific and used as received. The yields of the compounds reported here refer to the yields of spectroscopically pure compounds after purification. ^{1}H NMR spectra were recorded at 400 MHz on a Bruker ADVANCE 400 machine.

2. Preparation of gelatin polyzymes:

Polyzymes (**Fe_PZ**) were prepared through emulsification of a mixture of hemin and riboflavin in carvacrol into an aqueous gelatin solution, followed by irradiation with 365 nm UV-light. Briefly, hemin (17.2 mg/ml) and riboflavin (1 mg/ml) were solubilized in eugenol. Next, 3 μL of the resulting oil mixture was added to the gelatin aqueous solution (497 μL, 240 mg/L). This solution was then emulsified for 50 seconds using an amalgamator. The emulsion was then exposed to UV lamp for 20 minutes. The concentration of this polyzyme stock solution was defined as 100 v/v% (240 mg/L of gelatin).

3. Size of polyzymes

Hydrodynamic diameter of the polyzyme was measured by dynamic light scattering (DLS) in saline phosphate buffer (PBS, pH=7.4), using a Malvern Zetasizer Nano ZS instrument. The measurement angle was 173° (backscatter). Data were analyzed by the “multiple narrow modes” (high resolution) based on non-negative-least-squares (NNLS).

Transmission electron microscopy (TEM) samples of polymers were prepared by placing one drop of the desired solution on to a 300-mesh Cu grid-coated with carbon film. These samples were analyzed and photographed using JEOL CX-100 electron microscopy.

4. Quantification of hemin loaded in polyzymes

The quantification of Fe catalysts per particles were carried out by first drying 100 μL polyzyme (240 mg/L) under flowing nitrogen. Then the sample was redissolved in 3mL of THF and filtered through a PTFE syringe filter. The amount of loaded hemin was then quantified by UV-Vis, providing an average catalyst content of 0.43 mg per 26.5 mg eugenol per mg of gelatin. Standard hemin solutions were prepared for constructing calibration curve.

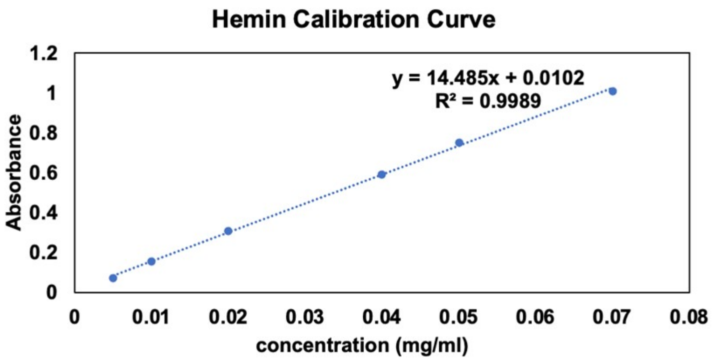

Supplementary Figure S1. Calibration curve of hemin solution.

5. Catalytic activity of Free Hemin catalysts

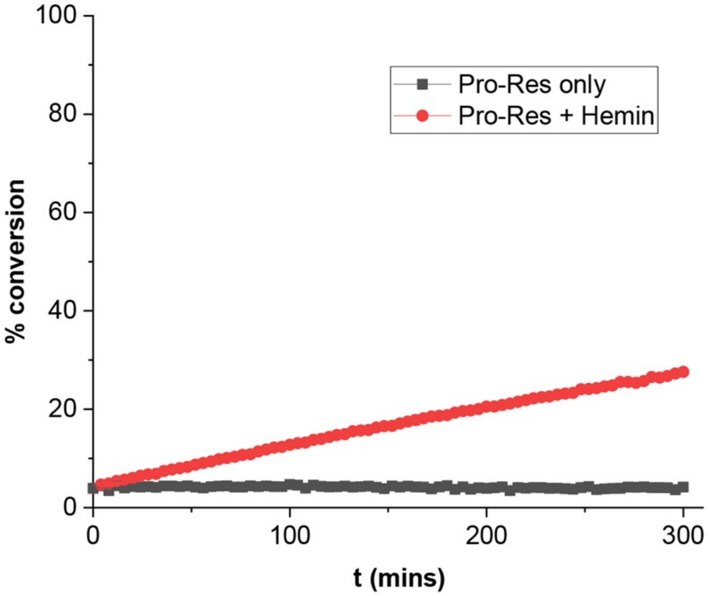

Supplementary Figure S2. Catalytic activity of Hemin was tracked by measuring changes in fluorescence (ex. 560 nm, em. 590 nm) of pro-resorufin solutions over time. Glutathione (GSH, 1 mM) was used as a cofactor for redox cycling.

6. Kinetic behavior of gelatin-based polyzymes

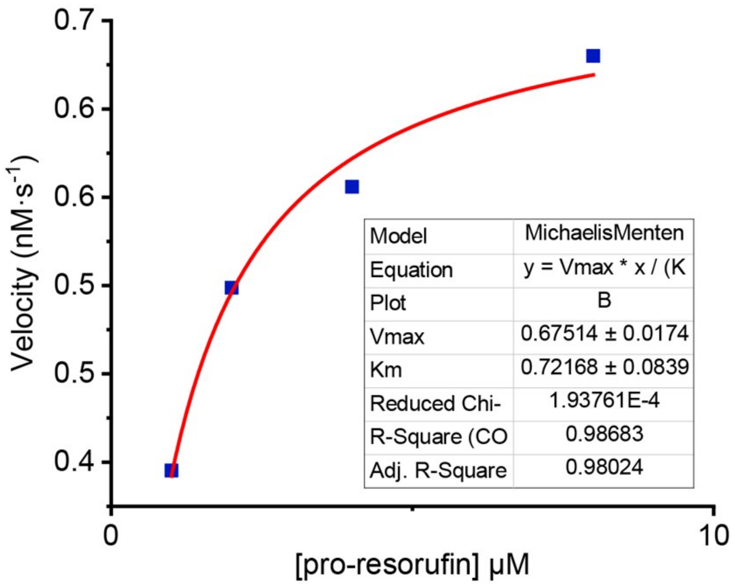

Supplementary Figure S3. The kinetics of polyzymes is shown as a function of substrate concentration; the line is the regression curve corresponding to Michaelis−Menten kinetics.

7. Minimum biofilm inhibitory concentration (MBIC) of Cip and pro-Cip

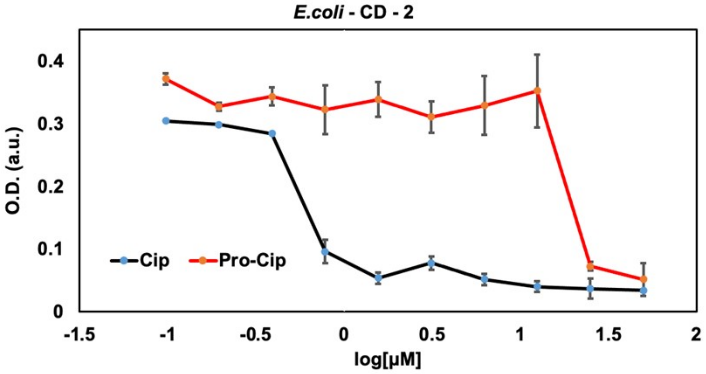

Supplementary Figure S4: minimum biofilm inhibitory concentration (MBIC) of **Cip** and **pro-Cip** toward *E. coli* (CD-2). The results showed that **pro-Cip** was less efficient to inhibit formation of CD-2 biofilm. We used established protocol with minor modification to test MBICs.[1] Briefly, *E. coli* bacteria (CD-2) were inoculated at 37 ^{o}C in TSB broth (2.5 mL/tube) until 0.5 McFarland standard. Then, 150 μL of this solution was seeded onto each well of a 96 well plate with pegged lid covered and cultured in a shaker at 50 rpm for 5 hours at 37 ^{o}C. Upon completion, the pegged lid was washed by transferring it onto a plate containing 200 μL PBS for 30 secs. The lid was then introduced onto another plate containing 200 μL of **pro-Cip** or **Cip** solution dissolved in M9 medium and incubated for 22 hours at 37 ^{o}C. MBICs were measured using optical density at 600 nm. M, molarity.

8. Biocompatibility of polyzyme and prodrug in mammalian cells

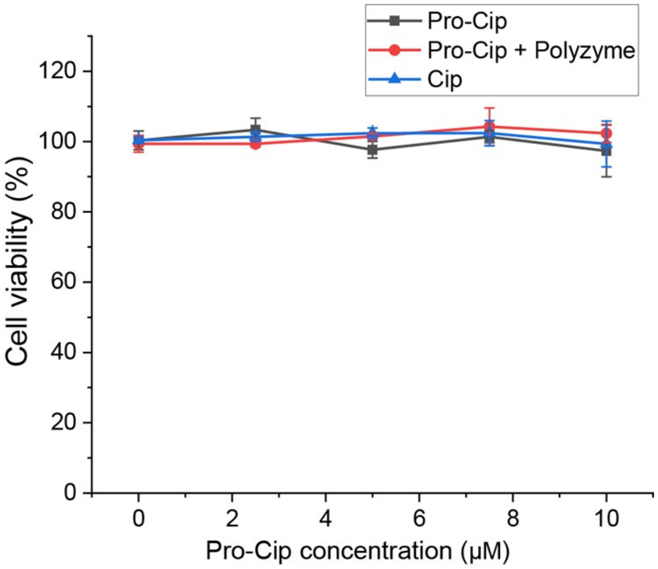

Supplementary figure S5. Viability of 3T3 fibroblast cells after 6 h treatment with different concentrations of **pro-Cip**, combination of **pro-Cip** and **Fe_PZ** (4.8 mg/L) or **Cip**. The data shown are average of triplicates, and the error bars indicate standard deviation.

9. Activation of pro-dye and prodrug in bacterial biofilms

For the confocal studies, GFP - *E. coli* seeding solutions were spiked with IPTG (Isopropyl β-D-1-thiogalactopyranoside) so that the final solution contained 1 mM of IPTG. 1 mL of the seeding solution was placed in confocal dishes and incubated at room temperature for 3 days to grow biofilms. M9 minimal media was replaced each day. Biofilms were washed with PBS three times on day 3 and incubated with 4.8 mg/L polyzyme in M9 media for 1 h. Bacterial biofilms were next washed three times with PBS to remove excess polyzyme and incubated with **pro-Res** (5 μM) for ~1 hr before obtaining images.

For the biofilm viability studies, 100 μL of *E. Coli* (CD-2) seeding solutions was added into each well of a 96 well-plate and incubated overnight at room temperature to grow the biofilms. The biofilms were then washed thrice with PBS and treated with the polyzyme (4.8 mg/L) in M9 media for 2 hrs. Next, bacterial biofilms were washed three times with PBS and treated with **pro-Cip**/**Cip** at varied concentrations prepared in M9 media. Bacterial biofilms were then incubated at 37 ^{o}C for 6 hrs. After this treatment, the biofilms were washed with PBS three times and their viability was determined using the alamar Blue assay according to the protocol established by the manufacturer.

10. Synthesis of pro-resorufin and pro-ciprofloxacin

The pro-resorufin and pro-ciprofloxacin were synthesized according to the previous protocol.[2]

1. Harrison, J. J.; Stremick, C. A.; Turner, R. J.; Allan, N. D.; Olson, M. E.; Ceri, H. Microtiter Susceptibility Testing of Microbes Growing on Peg Lids: A Miniaturized Biofilm Model for High-Throughput Screening. *Nat. Protoc*. 2010, **5**, 1236–1254.
2. Huang, R.; Li, C.-H.; Cao-Milán, R.; He, L. D.; Makabenta, J. M.; Zhang, X.; Yu, E.; Rotello, V. M. Polymer-Based Bioorthogonal Nanocatalysts for the Treatment of Bacterial Biofilms. *J. Am. Chem. Soc.* 2020, **142**, 10723–10729.
